# Characterization of Molecular Diversity and Organization of Phycobilisomes in Thermophilic Cyanobacteria

**DOI:** 10.3390/ijms24065632

**Published:** 2023-03-15

**Authors:** Jie Tang, Huizhen Zhou, Dan Yao, Lianming Du, Maurycy Daroch

**Affiliations:** 1 School of Food and Bioengineering, Chengdu University, Chengdu 610106, China; 2 School of Environment and Energy, Peking University Shenzhen Graduate School, 2199 Lishui Road, Shenzhen 518055, China

**Keywords:** thermophilic cyanobacterium, phycobiliprotein, phycobilisome, linker protein, lyase

## Abstract

Thermophilic cyanobacteria are cosmopolitan and abundant in the thermal environment. Their light-harvesting complexes, phycobilisomes (PBS), are highly important in photosynthesis. To date, there is limited information on the PBS composition of thermophilic cyanobacteria whose habitats are challenging for survival. Herein, genome-based methods were used to investigate the molecular components of PBS in 19 well-described thermophilic cyanobacteria. These cyanobacteria are from the genera *Leptolyngbya*, *Leptothermofonsia*, *Ocullathermofonsia*, *Thermoleptolyngbya*, *Trichothermofonsia*, *Synechococcus*, *Thermostichus*, and *Thermosynechococcus*. According to the phycobiliprotein (PBP) composition of the rods, two pigment types are observed in these thermophiles. The amino acid sequence analysis of different PBP subunits suggests several highly conserved cysteine residues in these thermophiles. Certain amino acid contents in the PBP of thermophiles are significantly higher than their mesophilic counterparts, highlighting the potential roles of specific substitutions of amino acid in the adaptive thermostability of light-harvesting complexes in thermophilic cyanobacteria. Genes encoding PBS linker polypeptides vary among the thermophiles. Intriguingly, motifs in linker *apcE* indicate a photoacclimation of a far-red light by *Leptolyngbya* JSC-1, *Leptothermofonsia* E412, and *Ocullathermofonsia* A174. The composition pattern of phycobilin lyases is consistent among the thermophiles, except for *Thermostichus* strains that have extra homologs of *cpcE*, *cpcF*, and *cpcT*. In addition, phylogenetic analyses of genes coding for PBPs, linkers, and lyases suggest extensive genetic diversity among these thermophiles, which is further discussed with the domain analyses. Moreover, comparative genomic analysis suggests different genomic distributions of PBS-related genes among the thermophiles, indicating probably various regulations of expression. In summary, the comparative analysis elucidates distinct molecular components and organization of PBS in thermophilic cyanobacteria. These results provide insights into the PBS components of thermophilic cyanobacteria and fundamental knowledge for future research regarding structures, functions, and photosynthetic improvement.

## 1. Introduction

Thermophilic cyanobacteria are ubiquitously distributed photosynthetic prokaryotes found in diverse thermal environments around the world [1,2]. In the past few decades, thermophilic cyanobacteria have been extensively explored as promising candidates for various applications related to agriculture, pharmaceutics, nutraceutical, and biofuel [3]. However, basic research and technological innovations are needed to fully explore the industrial potential of thermophilic cyanobacteria by thorough studies on each biological block of these organisms.

Phycobilisomes (PBS) are an important fragment of light-harvesting complexes present in most cyanobacteria. PBS capture light in regions of the visible spectrum and migrate energy to the photosystems [4]. Upon nitrogen starvation, the PBS also serve as a nitrogen storage and can be degraded to recover the nitrogen reserves [5]. The PBS complex is primarily composed of diverse, colored, and highly fluorescent phycobiliproteins (PBPs), while PBPs comprise several subunits, each formed by a protein backbone and a phycobilin linked by a covalent bond [6]. Based on long-wavelength absorption maxima, PBPs are divided into four major types: phycoerythrin (PE), phycocyanin (PC), phycoerythrocyanin (PEC), and allophycocyanin (APC) [7]. Four types of bilin chromophores are identified in cyanobacteria: phycoerythrobilin (PEB), phycocyanobilin (PCB), phycourobilin (PUB), and phycoviolobilin (PVB) [8]. PBPs and phycobilins from extreme environments have shown tremendous potential as antioxidant, anti-inflammatory anticancer, antimicrobial, antiplatelet, and antiviral compounds, and thus, could be applied in different industries, including food, feed, pharmaceutical, nutraceutical, and cosmetics [9]. More importantly, the PBPs from thermal environments may be more stable than that of mesophilic cyanobacteria (e.g., *Arthrospira platensis*, commonly known as Spirulina) or synthetic blue dyes (e.g., Brilliant Blue FCF) in a wider range of temperatures and pH conditions [10,11]. Therefore, it would be highly desirable to explore alternative sources for stable PBPs or to genetically modify the stability properties of PBPs in mesophilic species.

To date, PBS complexes in association with PSII are typically made up of two substructures: a core consisting of APC and a suite of rods arising out of the core, which may contain PC, PC, and PEC, and/or PE, depending on the species and growth environments [12]. A distinct rod-shaped PBS complex has also been described, which contains no APC but connects the rod by a unique CpcL linker protein directly to PSI [13,14]. Moreover, different groups of linker proteins and specific lyases for covalent attachment of the bilin chromophores also diversify the PBS structures [15]. Indeed, the composition of PBS varies from species to species, and individual organisms can have environmental acclimations due to external factors [7]. Obviously, understanding the molecular components of PBS is a prerequisite and will be useful for further research on structures, functions, biosynthesis, and downstream applications of a sort of microorganism from specific niches. In addition, molecular diversity can shed light on the evolution and structural characteristics (e.g., structural rigidity and thermostability) of PBPs [16,17]. The complexity of molecular components for PBS can also provide insights into metabolic engineering in mesophilic species since the heterologous production of mature PBPs is significantly challenging [18].

Recently, genomes of thermophilic cyanobacteria have been increasingly achieved by using next-generation sequencing (NGS). This affords an opportunity to rapidly investigate the molecular component of PBS in thermophilic cyanobacteria and the structural characteristics at the genomic level prior to biochemical studies. In the present study, we investigated the molecular basis of PBS by computational identification in the genome sequences of 19 thermophilic cyanobacteria. The function, evolution, and adaptations of these thermophilic cyanobacteria were further discussed in light of genetic diversity, sequence characteristics, and genomic organization of PBS components. The insights into the PBS components lay a solid foundation for future research regarding structures, functions, and photosynthetic improvement.

## 2. Results and Discussion

### 2.1. Genes Encoding PBPs in Thermophilic Cyanobacteria

Genes encoding phycobiliproteins, APC, PC, and PE, are shown in Table 1. All the studied thermophilic cyanobacteria possess one single set of *apcA* and *apcB* genes encoding α and β of APC. PBS core APC binds only the blue-colored chromophore phycocyanobilin (PCB), the biosynthesis gene (*pcyA*), which is also present in all the thermophiles with one single homolog (Table 1). Genes encoding PC and PE vary tremendously among these thermophiles. Two copies of α (*cpcA*) and β (*cpcB*) subunits of PC are present in *Leptolyngbya* JSC-1, *Leptothermofonsia* E412, and the six *Thermostichus* strains, compared to only one homolog of the two subunits in the other thermophilic cyanobacteria. Furthermore, *pebA* and *pebB* involved in the biosynthesis of phycocyanobilin (PEB) chromophore are present only in *Leptolyngbya* JSC-1 and *Leptothermofonsia* E412, suggesting that the remaining thermophilic cyanobacteria bind only to PCB and are of the C-PC type. Remarkably, PE-I encoded by *cpeA* and *cpeB* has been identified only in *Leptolyngbya* JSC-1 and *Leptothermofonsia* E412, whereas PE-II is absent in all the thermophiles. The absence of PE in these thermophiles suggests that PC might constitute the whole rod. Given the freshwater origins of these thermophilic cyanobacteria, such a high proportion of PE absence is consistent with the empirical knowledge that many freshwater cyanobacteria, e.g., *Synechocystis* PCC 6803 and *Synechococcus* PCC 7942, show no PE rods. As for the two PE-containing strains, the distal part of the PBS rods is probably composed of one type of PE (PE-I). Moreover, previous studies indicate that PE-I binds either only PEB or both PEB and phycourobilin (PUB) [19]. Herein, PE-I binds only PEB in the two thermophilic cyanobacteria in light of the absence of PUB-related biosynthesis genes (phycoerythrocyanin lyase/isomerase, *pecE*/*F*) [20]. According to the phycobiliprotein composition of the rods [21], the pigment types of the surveyed thermophilic cyanobacteria can be partitioned into two types: type 1 (T1) have only PC, and type 2 (T2) have PC and PE-I (Table 1).

Apart from α and β subunits of APC, all the thermophilic cyanobacteria also possess one homolog of *apcD* and *apcF* (Table S1), encoding the minor α-B and β-18 APC subunits, respectively. Both subunits have a lower abundance and replace α and β subunits in different APC trimers of membrane-contacted cylinders that form the core of the PBS [22]. Importantly, *apcD* and *apcF*, as well as the globular domain (PB domain) of the linker core–membrane (*apcE*), are indispensable for energy transfer to the photosystems [23].

### 2.2. Sequence Characteristics and Phylogenies of PBP Genes in Thermophilic Cyanobacteria

Functionally, each subunit of PBPs can have one to three phycobilin molecules attached to the polypeptide skeleton in highly preserved cysteine residues [24]. The amino acid sequences analysis of different PBP subunits in these thermophilic cyanobacteria suggests that there are several highly conserved cysteine residues, namely Cys-81 residues of APC subunits (Figure S1), Cys-85 residues of PC α subunit, Cys-83/110/154 residues of PC β subunit (Figure S2), Cys-82/139 residues of PE α subunit, and Cys-51/62/83/168 residues of PE β subunit (Figure S3). Notably, the Cys residue around the 81st site is conserved in all the PBP subunits of these thermophilic cyanobacteria, which is equivalent to the reported Cys-81 residue in other cyanobacteria that always show a bonded phycobilin [8].

Extensive comparisons of the amino acid content of each PBP polypeptide indicate that certain amino acid contents in the PBP of thermophiles are significantly higher than their mesophilic counterparts (Table 2). These results are consistent with previous reports that protein molecular adaptations to temperature are partially due to specific substitutions of amino acids, with glycine, serine, lysine, and asparagine in mesophiles, which are generally replaced in thermophiles by alanine, threonine, arginine, and glutamate, respectively [17,25], thus highlighting the potential roles of specific substitutions of amino acid in adaptive thermostability of light-harvesting complexes in thermophilic cyanobacteria. Interestingly, alanine is the most abundant amino acid category of PBP proteins in the studied thermophiles. Such alanine accumulation in PBP proteins may increase hydrophobicity and therefore decrease molecular flexibility, further enhancing protein thermostability to survive in thermal environments [26].

**Figure 1:**
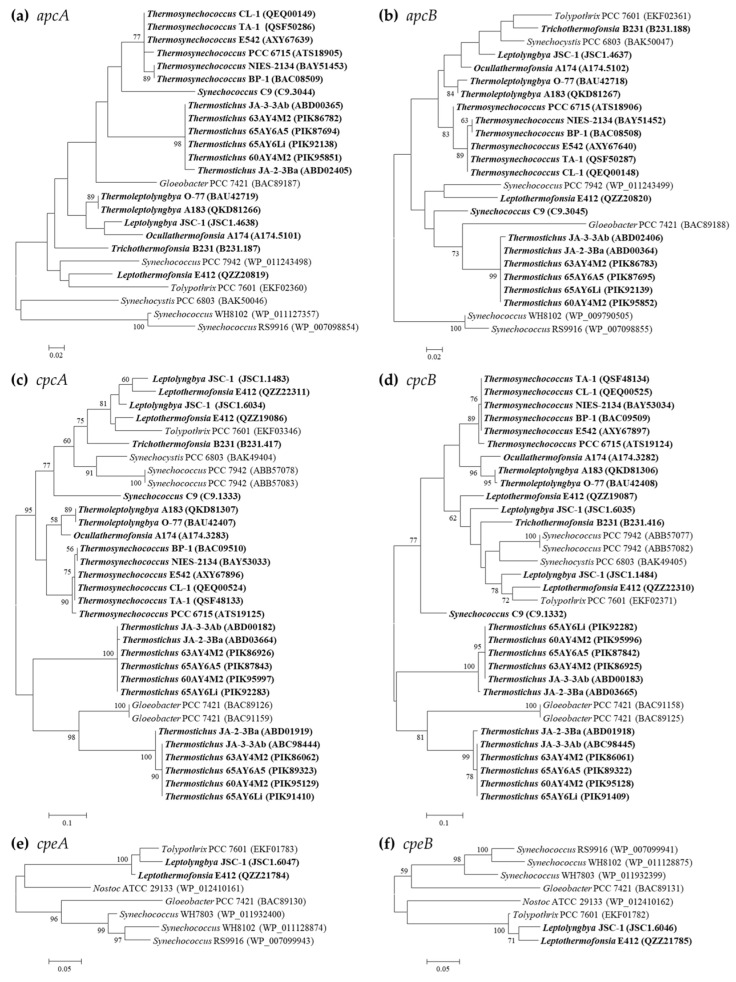
Phylogenetic inference of protein sequences encoding PBP subunits. The thermophilic cyanobacteria surveyed in this study are indicated in bold. Only bootstrap values > 50% are indicated at nodes. (**a**) *apcA*, (**b**) *apcB*, (**c**) *cpcA*, (**d**) *cpcB*, (**e**) *cpeA*, (**f**) *cpeB*.

Phylogenetic analyses suggest extensive genetic diversity in these PBP genes, as indicated by the assignments of these thermophilic cyanobacteria into different clusters or clades (Figure 1). Nevertheless, the grouping of the thermophilic strains from specific genus is consistent with the taxonomic lineages in all the phylograms, indicating high sequence conservation as revealed by extremely short branches (Figure 1). Among the thermophilic strains, a high degree of homology is noticed in *apcA* (>81.3%), *apcB* (>82.7%), *cpeA* (95.1%), and *cpeB* (83.6%) proteins, whereas the identity of *cpcA/B* proteins dramatically varies at the intergenus level, ranging from 53.0 to 93.8% and from 62.2 to 90.6%, respectively. In addition, unlike *Synechococcus* PCC 7942 and *Gloeobacter* PCC 7421 that harbor two identical copies of *cpcA/B* in the genome, the *cpcA* and *cpcB* homologs in the thermophiles are distinct, and are particularly divergent in *Thermostichus* strains (Figure 1c,d). Such a significant discrepancy of *Thermostichus* strains in the phylograms is further revealed by the low sequence conservation (*cpcA*: 60.3–60.9%; *cpcB*: 66.2–66.8%) between the corresponding opponents. However, domains analysis by PFAM tools suggests similar structures of *cpcA* or *cpcB* in *Thermostichus* strains. Experimental studies are required in future to elucidate the actual functions of the two copies of *cpcA/B* that differ in amino acid sequences. Moreover, the phylograms of *apcD* and *apcF* are in line with that of APC subunits (Figure S4).

Intriguingly, most of the thermophilic strains do not group with any non-thermophilic strains (Figure 1), indicating the specificity of PBP genes in the thermophiles. Exceptions are also noticed. The homologs of *cpcA/B* and *cpeA/B* within *Leptolyngbya* JSC-1 and *Leptothermofonsia* E412 cluster with that of freshwater filamentous *Tolypothrix* PCC 7601 (Figure 1c–f), showing high identities (>80%) to each other. Although the clustering of thermophilic strains does not completely comply with morphology, e.g., filamentous strains cluster with unicellular strains, the *apcA/B* and *cpeA/B* of marine strains (*Synechococcus* WH8102 and RS9916) are clearly discrepant to that of thermophilic and freshwater strains (Figure 1a,b,e,f), suggesting that habitats might be related to the genetic diversity of PBPs in cyanobacteria. However, future phylogenetic inferences of these molecular markers on a much larger scale may be helpful for the elaboration of the evolutionary relationship with cyanobacterial habitats and environments.

### 2.3. Genes Encoding PBS Linker Polypeptides in Thermophilic Cyanobacteria

The higher-order structure of PBS is stabilized by linker polypeptides that contribute to the building of a protein environment around the phycobilins [8]. Two types of APC-associated linker genes, *apcC* (core linker, L_C_) and *apcE* (core–membrane linker, L_CM_), are present in all the surveyed thermophilic cyanobacteria (Table 3). A gene copy of *apcE* varies among genera, while intragenus variation is evident only within the genus *Thermostichus*. Such an intragenus discrepancy may be brought about possibly by gene loss, recently gene acquisition, or limited duplication events that only occurred in certain strains.

**Figure 2:**
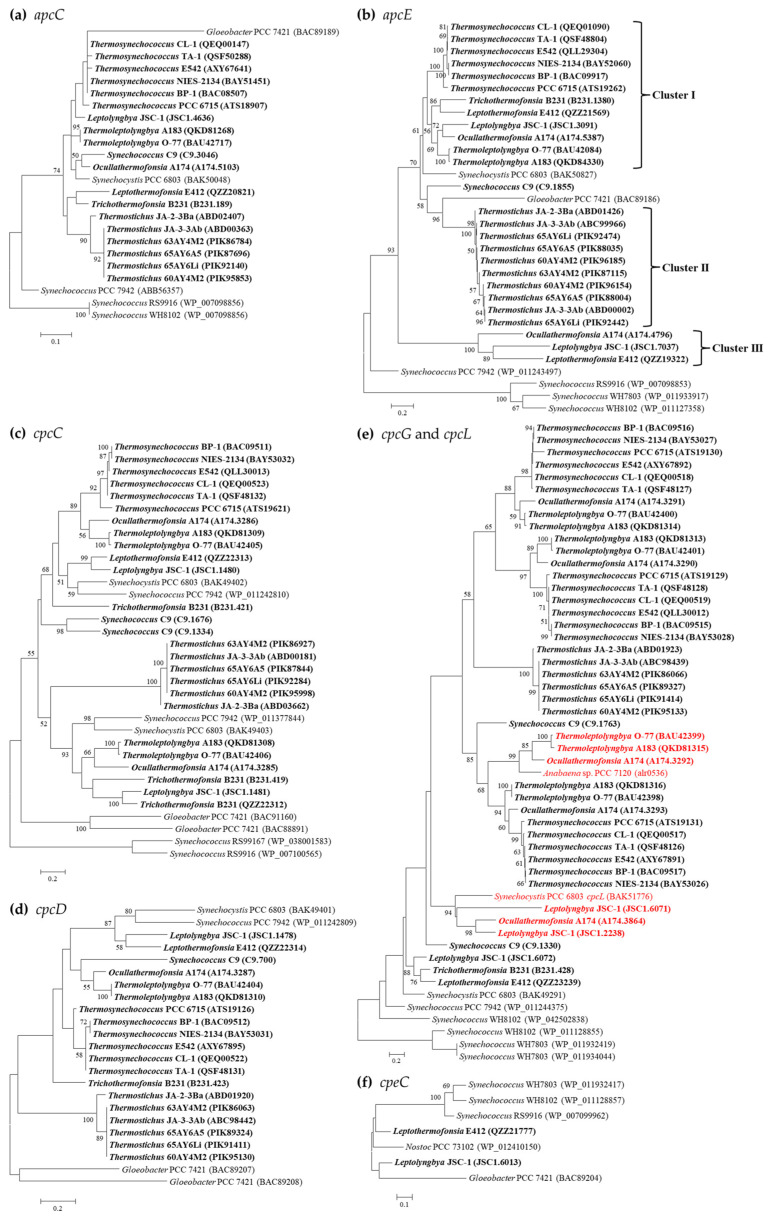
Phylogenetic inference of protein sequences encoding linker polypeptides. The thermophilic cyanobacteria surveyed in this study are indicated in bold. The putative *cpcL* is indicated in red. Only bootstrap values > 50% are indicated at nodes. (**a**) *apcC*, (**b**) *apcE*, (**c**) *cpcC*, (**d**) *cpcG* and *cpcL*, (**e**) *cpcD*, (**f**) *cpeC*.

Sequence analysis suggests a conserved pattern of *apcC* among the thermophilic cyanobacteria, exhibiting > 81% identities to that of *Synechocystis* PCC 6803. This is in accordance with the phylogram topology (Figure 2a). Remarkably, the sequence length of the *apcE* gene fluctuates from 778 (*Leptolyngbya* JSC-1) to 1139 (the six *Thermosynechococcus* strains) amino acids. The phylogram of *apcE* distributes the thermophilic cyanobacteria into three clusters (Figure 2b), which contain none of the non-thermophilic reference strains. Only *Synechococcus* C9 is an outlier and is solely located in a separate branch. Further domain analyses indicate that two predicted repeat (or linker-like) domains are possessed by *apcE* in strains belonging to cluster III, three in *Synechococcus* C9, *Trichothermofonsia* B231, and *Thermostichus* strains, and four domains in the remaining thermophiles (Figure 2b). The presence of additional L_CM_ domains in these strains suggests that their PBS core may have additional half-cylinders [27].

**Figure 3:**
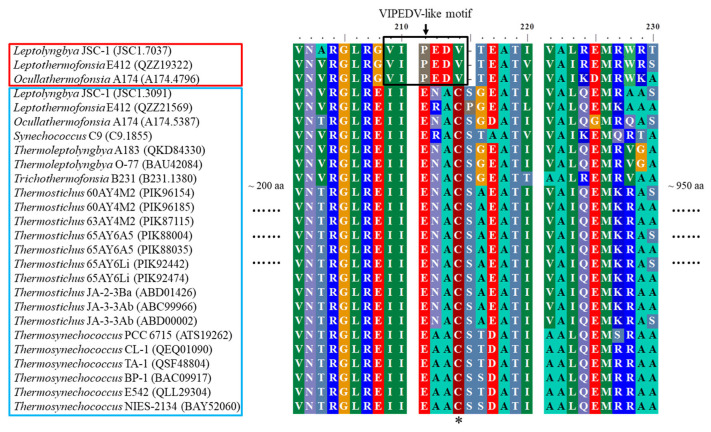
Conserved far-red specific-motifs present in *apcE* of thermophilic cyanobacteria. Thermophiles outlined in red contain conserved far-red-specific motifs in *apcE* sequences, while thermophiles in the blue box encompass conventional (white light) *apcE* sequences. The black box highlights the far-red-specific motif and the phytochrome-binding cysteine is indicated by an asterisk.

More interestingly, *Leptolyngbya* JSC-1, *Leptothermofonsia* E412, and *Ocullathermofonsia* A174 comprise two distinct *apcE* (Figure 2b). The amino acid sequences of *apcE* affiliated to cluster III show conserved VIPEDV-like motifs (Figure 3), which are identical to a recently identified far-red-specific area within *apcE2* of cyanobacteria [28]. This result suggests that the three *apcE* of thermophilic cyanobacteria encode a phycobilisome linker associated with FRL photosynthesis, meanwhile highlighting their capabilities of adjusting a photosynthetic apparatus through chromatic adaptation. The other, conventional, *apcE* sequences show a highly conserved phytochrome-binding cysteine (Figure 3) that is missing in far-red sequences. The missing cysteine in *apcE* implies the non-covalent binding of phycocyanobilin and might relate to an important red-shift in the absorbance (e.g., 700 nm in the far-red *Synechococcus* PCC 7335, as opposed to 660 nm for covalent binding) [29]. Photoacclimation of a far-red light by *Leptolyngbya* JSC-1 and *Leptothermofonsia* E412 has been experimentally verified [30,31], and the responses to the ambient light environment might be mediated by two-component systems involved in signal transduction [32]. Moreover, the *Ocullathermofonsia* strain might expand the diverse array of far-red photosynthesizing cyanobacteria.

Four types of PC-associated linker genes, *cpcC/D* (rod linker, L_R_), *cpcG* (rod–core linker, L_RC_), and *cpcL* (L_RM_, rod–membrane linker) have been identified in the surveyed thermophilic cyanobacteria (Table 3). Except for *cpcD*, intergenus variations are noticed in the *cpcC* and *cpcG* gene numbers. The six filamentous thermophiles and unicellular *Synechococcus* C9 harbor two *cpcC* genes, while the other unicellular thermophiles contain only one homolog. The *cpcC* phylogram indicates that the two homologs from the same strain are quite distinct and all the thermophilic cyanobacteria are divergent from non-thermophilic reference strains, as suggested by long branches (Figure 2c). Identity calculation reveals a low sequence conservation of *cpcC* homologs between thermophilic cyanobacteria and reference *Synechococcus* PCC 7942 (41.4–63.3%). Nevertheless, domain analysis suggests a similar structure of *cpcC* between all the thermophilic cyanobacteria and *Synechococcus* PCC 7942. In addition, the presence of *cpcC/D* in *Leptolyngbya* JSC-1 and *Leptothermofonsia* E412 is contrary to the previous findings in marine *Synechococcus* strains that PE-I-containing strains possess none of the *cpcC* and *cpcD* homologs [21,33]. Taken together, our results indicate that cyanobacteria strains belonging to a given pigment type may own different structures of PBS rods. Moreover, for the case with multiple *cpcC*, determining which *cpcC* is located in a central position in the PC rod or in a peripheral position requires future experimental study, e.g., using rod mutants [34]. Similar to *cpcC*, phylogenetic analysis and identity calculation suggest a relatively low sequence conservation of *cpcD* homologs as revealed by long branches (Figure 2d) and low identities to *Synechococcus* PCC 7942 (30.0–50.6%), and similar domain structures are observed.

As for *cpcG*, the homolog number tremendously varies among thermophilic cyanobacteria, from one to three (Table 3). The phylogram of CpcG proteins (Figure 2e) indicates that they are categorized into subgroups, which appears to fit within the structural variations of the phycobilisome core. For example, the multi-type of *cpcG* genes in *Thermosynechococcus* strains may suggest a large pentacylindrical core [35], while the *Thermostichus* strains, such as *Synechococcus* PCC 7942, may correspond to a small bicylindrical core due to the single *cpcG* gene [36]. These indicate that various CpcG proteins have evolved to play a specific role in the assembly of the rods with diverged core structures. In addition, six homologs from *Leptolyngbya* JSC-1, *Ocullathermofonsia* A174, *Thermoleptolyngbya* A183 and O-77 cluster, with the *cpcL* of *Synechocystis* PCC 6803 [37] and *Anabaena* PCC 7120 [14], respectively (Figure 2e). A hydrophobic segment is also characterized in the C-terminal region of these homologs (Table S2), which differs from the hydrophilic sequence of *cpcG* that associates with the APC core [38]. Taken together, these homologs from thermophilic cyanobacteria could be putative *cpcL*, which may anchor *cpcL*-PBS to the thylakoid membrane or PSI complex [37]. Moreover, this result suggests that these *cpcL*-owned cyanobacteria may utilize CpcL–PBS as an alternative form to harvest energy in association with PSI. Moreover, the phylogram reveals that the *cpcL* clusters are mixed with *cpcG* clusters in a large phylogenetic clade. This implies that the acquisition or loss of the hydrophobic C-terminal transmembrane helix may occur frequently, enabling cyanobacteria to flexibly alter light energy distribution to the photosystems via the domain reorganization of CpcG and CpcL family proteins [13]. Herein, whether the sequence discrimination of these *cpcL* genes results in any biological function shift needs further careful investigations. Intriguingly, *Ocullathermofonsia* A174 possesses two types of *cpcL*, and what the actual functions of the two *cpcL* are also requires further investigations.

Only one putative PE-associated linker gene, *cpeC* (L_R_), is present in the two PE-I-containing thermophilic strains (Table 3). Of the *cpeC* phylogram (Figure 2f), *Leptolyngbya* JSC-1 and *Leptothermofonsia* E412, together with another freshwater cyanobacterium, *Nostoc punctiforme* PCC 73102, form a distinct clade apart from the clade composed of marine *Synechococcus* strains, both of which are supported by robust bootstrap values. This result suggests that *cpeC* may be subjected to different evolution related to cyanobacterial habitats and environments.

### 2.4. Genes Encoding Phycobilin Lyases in Thermophilic Cyanobacteria

**Figure 4:**
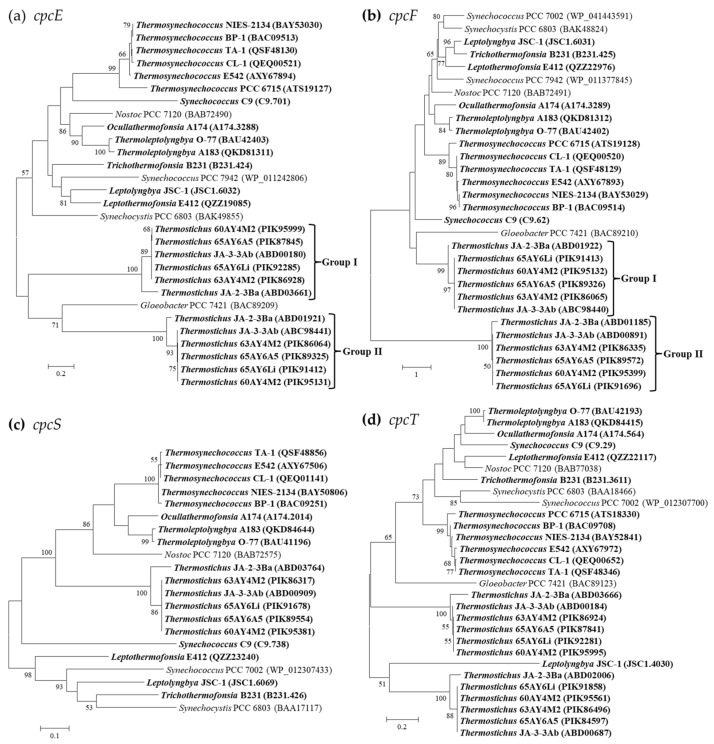
Phylogenetic inference of protein sequences encoding PC-associated phycobilin lyases. The thermophilic cyanobacteria surveyed in this study are indicated in bold. Only bootstrap values > 50% are indicated at nodes. (**a**) *cpcE*, (**b**) *cpcF*, (**c**) *cpcS*, (**d**) *cpcT*.

Three families or clans of phycobilin lyases (*E*/*F*, *cpcS*/*cpcU*, and *T* families), enzymes involved in the chromophorylation of phycobiliproteins, have been described to date based on phylogenetic, structural, and biochemical studies (respective substrates and enzymatic activities) [39]. Four types of PC-associated phycobilin lyases have been identified in the thermophilic cyanobacteria (Table 4). One homolog of *cpcE*/*F* genes encoding a heterodimeric complex are present in all the surveyed thermophilic cyanobacteria, except for *Thermostichus* strains, which have an additional homolog (Table 4). Though the sequence differences of *cpcE*/*F* are evident, as suggested by the long branches (Figure 4a,b), most of the thermophilic cyanobacteria cluster with the reference strain *Nostoc* sp. PCC 7120, whose crystal structure of *cpcE*/*F* is primarily α-helical, consisting of crescent-shaped elongated superhelices or solenoids [40]. Only the two homologs of *Thermostichus* strains form two distinct clades. Further domain analysis suggests that such a discrepancy might be mainly ascribed to the existence of multiple HEAT-repeat or HEAT-like-repeat domains that are both assigned to clan CL0020. Among the *cpcE* gene, one HEAT-repeat and one HEAT-like-repeat domain are present in the six filamentous thermophiles and *Synechococcus* C9. Two types of domain patterns are present in the six *Thermosynechococcus* strains, namely one HEAT-repeat and one HEAT-like-repeat domain (BP-1 and NIES-2134), and two HEAT-repeat and one HEAT-like-repeat domains (PCC 6715, CL-1, TA-1, and E542). As for the two distinct homologs of *cpcE* in *Thermostichus* strains, group I (Figure 4a) contains one HEAT-repeat domain, while two HEAT-repeat and one HEAT-like-repeat domains are shown by group II, except for JA-2-3Ba, which comprises one HEAT-repeat and one HEAT-like-repeat domain.

Similarly, variations of the HEAT-repeat and HEAT-like-repeat domains exist in the *cpcF* gene among the thermophilic cyanobacteria. One HEAT-repeat and one HEAT-like-repeat domain are present in *Ocullathermofonsia* A174, while the other five filamentous thermophiles and *Synechococcus* C9 contain only one HEAT-repeat domain. *Thermosynechococcus* PCC 6715 comprises one HEAT-repeat domain, whereas the remaining *Thermosynechococcus* strains possess one HEAT-repeat and one HEAT-like-repeat domain. One HEAT-repeat and one HEAT-like-repeat domains are present for both the two homologs of *cpcF* in *Thermostichus* JA-2-3, while the other *Thermostichus* strains show a different pattern of domains, including one HEAT-repeat for group I and three HEAT-repeat domains for group II. Moreover, the number of HEAT-repeat and HEAT-like-repeat domains insignificantly matched to the Pfam database differs among the *cpcE*/*F* of thermophilic cyanobacteria, also contributing to the sequence divergences as indicated by the phylograms (Figure 4a,b). The *cpcE/F* genes in the thermophilic cyanobacteria might be involved in ligating chromophores to the α-subunits of phycobiliproteins and the HEAT-repeat motifs within them may facilitate protein–protein interactions [41,42].

Homolog of *cpcS* is present in all the thermophilic cyanobacteria except for *Thermosynechococcus* PCC 6715 (Table 4). Such an absence of *cpcS* may be caused by gene loss. Although the phylogram suggests the classification of these strains into several divergent clusters (Figure 4c), a similar domain architecture is shared by all the cyanobacteria studied. Two homologs of *cpcT* are shown in *Thermostichus* strains, while the other thermophilic cyanobacteria possess only one. In addition, the two homologs in *Thermostichus* strains are distinct from each other, as indicated in the phylogram (Figure 4d). However, domain analysis again confirms a consistent pattern for this lyase.

**Figure 5:**
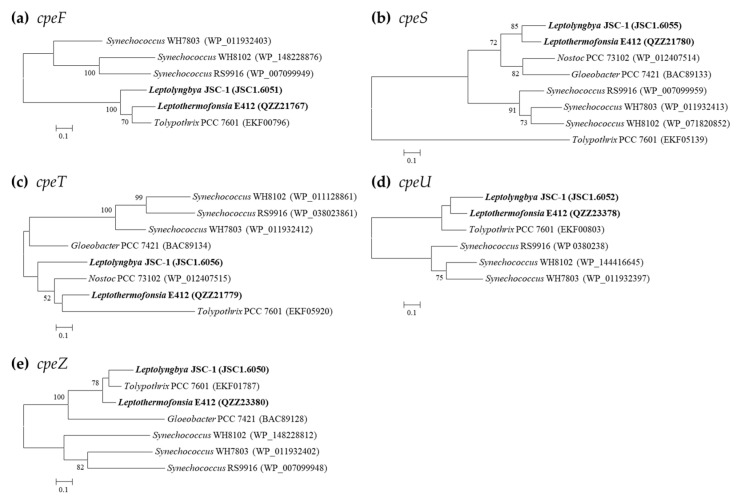
Phylogenetic inference of protein sequences encoding PE-associated phycobilin lyases. The thermophilic cyanobacteria surveyed in this study are indicated in bold. Only bootstrap values > 50% are indicated at nodes. (**a**) *cpeF*, (**b**) *cpeS*, (**c**) *cpeT*, (**d**) *cpeU*, (**e**) *cpeZ*.

PE-associated phycobilin lyases are only in possession of *Leptolyngbya* JSC-1 and *Leptothermofonsia* E412 (Table 4), showing a consistent pattern of gene category and number. The two thermophiles exhibit a high degree of homology (71.9–78.2%) with the *cpeF* of filament *Tolypothrix* PCC 7601, an experimentally confirmed bilin lyase that is responsible for the attachment of the doubly ligated PEB to Cys-48/Cys-59 of *cpeB* [43]. The clade formed by the three strains is divergent from the marine *Synechococcus* strains, as revealed by the *cpeF* phylogram (Figure 5). The phylograms of *cpeU* and *cpeZ* are congruent with the *cpeF* phylogram, whereas the *cpeS* and *cpeT* of the two thermophilic cyanobacteria appear to be divergent from that of *Tolypothrix* PCC 7601 (Figure 5). Homologs of *cpeZ* in the two thermophiles may function as chaperones, facilitating *cpeF* and *cpeB* interaction by stabilizing *cpeB*s conformation [44]. Moreover, homologs of *cpeS*, *cpeT*, and *cpeU* in the two thermophiles may attach PEB to Cys residues of the *cpeB* subunit of PE [44,45].

Overall, all the specific bilin lyases might ensure the binding of the correct bilin to the corresponding cysteine residue with the correct stereochemistry, finally contributing to the post-translational modification and assembly of PBP in vivo into mature light-harvesting complexes. Future experimental studies should be carried out to elucidate how these bilin lyases chromophorylate the PEB-binding sites on PBP in the thermophilic cyanobacteria using, e.g., biochemical assays and site-directed mutagenesis.

### 2.5. Genomic Distribution Pattern of PBS-Related Genes in Thermophilic Cyanobacteria

**Figure 6:**
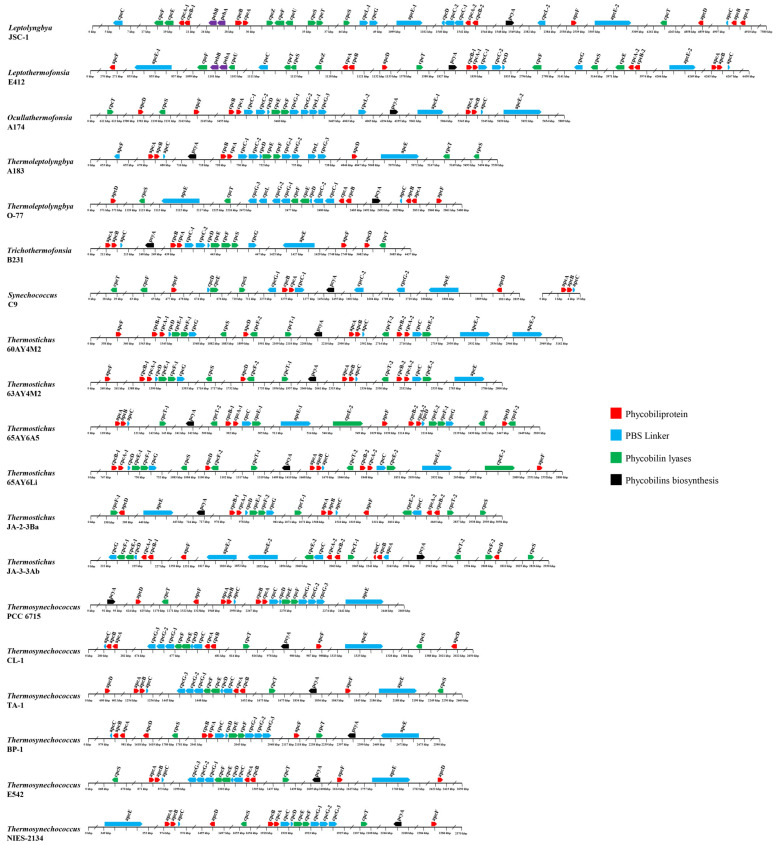
Genomic organization of PBS-related genes in the 19 thermophilic cyanobacteria surveyed. Solid arrow boxes refer to genes and the direction of transcription.

The genomic organization of PBS-related genes in the genomes of the surveyed thermophilic cyanobacteria may provide useful insights into the function and evolution of these genes. Herein, the genomic organization of PBS-related genes is graphically presented in Figure 6. Generally, the comparative genomic analysis suggests diversified molecular components and the organization of PBS-related genes among the surveyed thermophilic cyanobacteria, which is especially evident at the genus level. Moreover, the different genomic distribution of PBS-related genes among the thermophiles probably indicates various regulations of expression. Intriguingly, the distribution of PBS rod-related genes in these thermophiles is quite divergent from that of marine *Synechococcus* representatives with all known pigment types. The PBS rod-related genes for marine *Synechococcus* strains of different pigment types are primarily located in a dedicated genomic region, which is oriented from the phenylalanine tRNA (left) to the conserved low molecular weight tyrosine phosphatase *ptpA* (right) [33]. No such distributions are noticed in the thermophiles. It is not surprising that freshwater *Synechococcus* C9 shows a distinct distribution pattern to marine *Synechococcus* strains, since the latter are phylogenetically divergent from the former [46]; recently, a revision of its name to *Parasynechococcus* has been proposed.

A similar distribution pattern of APC-associated genes is shared by all the genomes of the thermophiles (Figure 6). Briefly, small groups cluster together with three APC core genes, in the order *apcA-B-C*, while three other core genes, *apcD*, *apcE*, and *apcF*, have no APC genes in their close vicinity. This result indicates that in these thermophiles, *apcC* might be co-regulated with *apcA/B*, while *apcD*, *apcE*, and *apcF* may be independently regulated. The standalone regulation of *apcD* and *apcF* might be important for adjusting energy transfer and state transitions, since the role of these proteins could be different in different strains [23]. The cluster in these thermophiles is different from the *apcE-A-B-C* cluster of marine *Synechococcus* strains [21], suggesting distinct regulations of the core–membrane linker. The expression of *apcE* in a locus remote from the core operon may provide the thermopiles with the flexibility of the PBS assembling, energy transfer to PSII, and photoprotection [47].

Most of the PBS rod genes are located in a much larger cluster, the size of which increases with the complexity of the rod structure (Figure 6). For the PC-associated genes, several distribution patterns are observed in the genomes of the thermophiles. First, a large cluster of PC-associated genes is found in the genomes of *Ocullathermofonsia* A174, *Thermoleptolyngbya* A183 and O-77, *Trichothermofonsia* B231, and the six *Thermosynechococcus* strains. The cluster is in the order *cpcB-A-C-D-E-F-G*, within which the number of *cpcC* and *cpcG* homologs varies from one to two and from one to three, respectively. Only *cpcS* and *cpcT* positionally disperse from the core cluster. The large cluster may reveal the co-regulation of these genes and the core components of the PC complex. Nevertheless, the cases in *Ocullathermofonsia* A174 and *Thermoleptolyngbya* A183 and O-77 might be more complex. A structure of *cpcG1-cpcG2-cpcL-cpcG3* is observed in the three strains, which is consistent with the operon in *Anabaena* PCC 7120. The conventional PBS of *Anabaena* PCC 7120 only contains the products of CpcG1, CpcG2, and CpcG3 [14]. Therefore, regulation of *cpcL* for the rod-shaped PBS may be different from the regulation of the large gene cluster. The functional role of *cpcL* in acclimation to changes in light conditions has been reported to be widely distributed in cyanobacteria [13,48], indicating the plastic regulation of *cpcL* expression in response to changes in environmental conditions. Second, the genomes of *Thermostichus* strains comprise two clusters of PC-associated genes, namely *cpcB-A-D-E-F g* and *cpcB-A-C-E*. One homolog of *cpcT* is positionally close to the latter cluster but shows an opposite transcription direction. Furthermore, a homolog of *cpcE*, *-F*, *g*, and -*T* is also found to be distant from the two clusters. Third, the distribution pattern of PC-associated genes is complex in *Leptolyngbya* JSC-1 and *Leptothermofonsia* E412. Both genomes possess a cluster in the order of *cpcB-A-C-C-D*, whereas clusters *cpcB-A-E-F* and *cpcB-A-E* are present in JSC-1 and E412, respectively. The other PC-associated genes exhibit scattered distribution in the two genomes. Fourth, *Synechococcus* C9 shows a distinct distribution pattern of PC-associated genes, comprising two small clusters (*cpcB-A-C* and *cpcD-E*) and dispersal distribution of the other genes. Additional to the clusters in each genome, the isolated lyase genes, e.g., *cpcS* and *cpcT*, may flexibly modulate both the chromophore binding and detachment in these thermophiles to optimize light harvesting of the PBS to changing light environments [49].

The vast majority of PE-associated genes in the genomes of *Leptolyngbya* JSC-1 and *Leptothermofonsia* E412 are located in a large cluster ranging from 8 to 10 kbp, while only *cpeC* in JSC-1 and *cpeT* and *cpeU* in E412 are the outliers of this cluster (Figure 6). Although PE-associated genes are widely distributed in a large region, they may directly or indirectly regulate each other. For instance, the *cpeT* mutant strain of *Tolypothrix* PCC 7601 showed the downregulation of *cpeB-A* and the upregulation of the *cpeCDESTR* operon, suggesting potential regulatory roles of *cpeT* on the expression of these genes in addition to its role as a PEB lyase for chromophorylation on the β-subunit of PE [45]. The chromophorylation efficiency of CpeT can be improved with the help of the chaperone-like protein CpeZ, which can also facilitate the lyase activity of CpeS, CpeT, and CpeU [43,50]. Thus, these studies imply the underlying regulatory network of these PE-associated genes in the two thermophilic cyanobacteria. Taken together, the two thermophiles exhibit a sophisticated distribution pattern of PC and PE-associated genes and that chromatic acclimation can be regulated to adapt to different light conditions.

## 3. Materials and Methods

### 3.1. Genome Collection of Thermophilic Cyanobacteria

Cyanobacteria with available genomes and closely related to thermophilic or hot-spring strains were retrieved as a preliminary dataset from the genomic resources of the NCBI at the time of this study (1 June 2022). Further, the rigid collection of thermophilic cyanobacteria was verified and retained based on the literature searches and validation of thermophilic characteristics through contacting culture collections, leading authors of the manuscripts, and submissions. Then, quality control for all genomes was performed using the following criteria to reduce data redundancy and biased genome representation of cyanobacteria. First, genomes with average nucleotide identity (ANI, calculated using an online tool: http://enve-omics.ce.gatech.edu/ani/, accessed on 20 July 2022) values greater than 99.9% were considered as redundant genomes, and then only one of these genomes was randomly kept for the analysis. Second, the quality of the genomes was evaluated using CheckM [51] to ensure a more reliable genome dataset with near completeness (>98%) and low contamination (<2%). Finally, a dataset comprising 19 thermophilic cyanobacteria was established (Table S3). The genome, protein sequences, and genomic annotations of the thermophilic cyanobacteria studied were retrieved from the database of NCBI. The genomes with no or incomplete annotations were annotated using the RAST annotation system [52], which is provided in Table S4.

Detailed information regarding ecology, morphology, and genome characteristics of the 19 thermophilic strains was summarized in Table S3. Briefly, the 19 thermophiles were taxonomically affiliated to six families of order Pseudanabaenales, Synechococcales, and Thermostichales, including Leptolyngbyaceae: *Leptolyngbya* sp. JSC-1 [53] and *Leptothermofonsia sichuanensis* E412 [31]; Oculatellaceae: *Ocullathermofonsia sinensis* A174, *Thermoleptolyngbya* sp. O-77 [54], and *T. sichuanensis* A183 [55]; Synechococcaceae: *Synechococcus* sp. C9 [56]; Thermostichaceae: *Thermostichus* sp. 60AY4M2, 63AY4M2, 65AY6A5, 65AY6Li [57], JA-2-3B, and JA-3-3Ab [58]; Thermosynechococcaceae: *Thermosynechococcus lividus* PCC 6715 [59], *Thermosynechococcus* sp. CL-1 [60] and TA-1 [61], *T. vestitus* BP-1 [62], and E542 [63], *T. vulcanus* NIES-2134 [16]; and Trichocoleusaceae: *Trichothermofonsia sichuanensis* B231.

### 3.2. Identification of Orthologous Proteins

Amino acid sequences of proteins involved in PBS of *Synechocystis* sp. PCC 6803, and/or *Synechococcus* sp. PCC 7942, and/or *Synechococcus* sp. WH8102 were downloaded from the CyanoBase (http://genome.kazusa.or.jp/cyanobase, accessed on 20 August 2022) or BioCyc (https://biocyc.org/, accessed on 22 August 2022) as a reference protein set. Based on the bidirectional best hit (BBH) criterion [64], orthologous proteins involved in PBS of the surveyed thermophilic species were identified using BLASTP with the following thresholds: E-value cut-off of 1E-6, ≥30% identity, and 70% coverage. The identified proteins were homologous to these proteins in the three aforementioned species, namely PBP (*apcA*, *apcB*, *apcD*, *apcF*, *cpcA*, *cpcB*, *cpeA*, *cpeB*, *mpeA*, *mpeB*, *rpcA*, and *rpcB*,), PBS linkers (*apcC*, *apcE*, *cpcC*, *cpcD*, *cpcG*, *cpeC*, *cpeF*, *mpeC*, *mepD*, *mpeE*, *mpeF*, and *mpeG*), phycobilin lyases (*cpcE*, *cpcF*, *cpcS*, *cpcT*, *cpeF*, *cpeS*, *cpeT*, *cpeU*, *cpeY*, *cpeZ*, *rpcE*, *rpcF*, *rpcG*, *rpcT*, *mpeU*, *mpeV*, *mpeY*, and *mpeZ*), and phycobilin biosynthesis (*pcyA*, *pebA*, *pebB*, *pebS*, *pecE*, *pecF*, *rpcG*, *pecE*, and *pecF*). The accession numbers of orthologous proteins identified in each genome of the thermophilic cyanobacteria studied were summarized in Table S1.

### 3.3. Protein Sequence Analysis

Amino acid sequences were collected for the surveyed thermophilic species and reference cyanobacteria to reconstruct phylogenies. Multiple sequence alignments were performed by muscle complemented in Mega7 [65]. Maximum-likelihood (ML) phylogenetic analyses were carried out using PhyML v3.0 [66], and the substitution models were automatically selected by the model selection function implemented in PhyML [67] under Bayesian information criterion (BIC). Parameter settings in PhyML and bootstrap analysis of phylogenies were followed as described [68].

Domains within protein sequences were identified by the PFAM tool of European Bioinformatics Institute (EMBL-EBI) (http://pfam-legacy.xfam.org/, accessed on 20 July 2022), and domain architectures were further viewed using the HMMER webserver [69].

## 4. Conclusions

In the present study, the molecular components of PBS in 19 well-described thermophilic cyanobacteria are elucidated using genome-based methods. According to the PBP composition of the rods, two pigment types are observed in the surveyed thermophiles. The amino acid sequence analysis of different PBP subunits suggests several highly conserved cysteine residues in these thermophiles. Certain amino acid contents in the PBP of thermophiles are significantly higher than their mesophilic counterparts, highlighting the potential roles of the specific substitutions of amino acids in the adaptive thermostability of light-harvesting complexes in thermophilic cyanobacteria. The genes encoding PBS linker polypeptides vary among the thermophiles. Intriguingly, motifs in linker *apcE* indicate the photoacclimation of a far-red light by *Leptolyngbya* JSC-1, *Leptothermofonsia* E412, and *Ocullathermofonsia* A174. The composition pattern of phycobilin lyases is consistent among the thermophiles, except for *Thermostichus* strains, which have an extra homolog of *cpcE*, *cpcF* and *cpcT*. In addition, phylogenetic analyses of genes coding for PBPs, linkers, and lyases suggest extensive genetic diversity among these thermophiles. Moreover, the different genomic distribution of PBS-related genes among the thermophiles probably indicates various regulations of expression. In summary, the comparative analysis elucidates the distinct molecular components and organization of PBS in thermophilic cyanobacteria. These results provide insights into the PBS components of thermophilic cyanobacteria and fundamental knowledge for future research regarding structures, functions, and photosynthetic improvement.

## Figures and Tables

**Table 1:** Species and PBP characteristics of thermophilic cyanobacteria studied.

Species	Morphology	Allophycocyanin		Phycocyanin		Phycoerythrin-I		Phycocyanobilin	Phycoerythrobilin		Pigment Type
		*apcA*	*apcB*	*cpcA*	*cpcB*	*cpeA*	*cpeB*	*pcyA*	*pebA*	*pebB*	
*Leptolyngbya* sp. JSC-1	Filamentous	1	1	2	2	1	1	1	1	1	T2
*Leptothermofonsia sichuanensis* E412	Filamentous	1	1	2	2	1	1	1	1	1	T2
*Ocullathermofonsia sinensis* A174	Filamentous	1	1	1	1			1			T1
*Thermoleptolyngbya sichuanensis* A183	Filamentous	1	1	1	1			1			T1
*Thermoleptolyngbya* sp. O-77	Filamentous	1	1	1	1			1			T1
*Trichothermofonsia sichuanensis* B231	Filamentous	1	1	1	1			1			T1
*Synechococcus* sp. C9	Unicellular	1	1	1	1			1			T1
*Thermostichus* sp. 60AY4M2	Unicellular	1	1	2	2			1			T1
*Thermostichus* sp. 63AY4M2	Unicellular	1	1	2	2			1			T1
*Thermostichus* sp. 65AY6A5	Unicellular	1	1	2	2			1			T1
*Thermostichus* sp. 65AY6Li	Unicellular	1	1	2	2			1			T1
*Thermostichus* sp. JA-2-3Ba	Unicellular	1	1	2	2			1			T1
*Thermostichus* sp. JA-3-3Ab	Unicellular	1	1	2	2			1			T1
*Thermosynechococcus lividus* PCC 6715	Unicellular	1	1	1	1			1			T1
*Thermosynechococcus* sp. CL-1	Unicellular	1	1	1	1			1			T1
*Thermosynechococcus* sp. TA-1	Unicellular	1	1	1	1			1			T1
*Thermosynechococcus vestitus* BP-1	Unicellular	1	1	1	1			1			T1
*Thermosynechococcus vestitus* E542	Unicellular	1	1	1	1			1			T1
*Thermosynechococcus vulcanus* NIES-2134	Unicellular	1	1	1	1			1			T1

Copy numbers of genes are indicated.

**Table 2:** Comparisons of amino acid content of each PBP polypeptide between thermophiles (the 19 thermophilic cyanobacteria investigated in this study) and mesophiles (*Synechocystis* PCC 6803 and *Synechococcus* PCC 7942).

PBP	Subunit	Amino Acid Content	Thermophiles	Mesophiles	*p* Value ^1^
APC	*apcA*	Glutamate	3.31 ± 0.35%	2.17 ± 0.31%	0.014
	*apcB*	Serine	6.34 ± 0.91%	9.01 ± 0.31%	0.020
CPC	*cpcA*	Glycine	7.45 ± 0.24%	8.00 ± 0.02%	0.022
	*cpcB*	Lysine	3.15 ± 0.47%	2.32 ± 0.58%	0.017

^1^ Statistical significance is calculated using Kruskal–Wallis test. Significance levels of 0.05 and 0.01 were applied for the analysis. Only data that are statistically significant are shown.

**Table 3:** Availability of genes encoding linker polypeptides in the genomes of thermophilic cyanobacteria studied.

Species	Pigment Type	Allophycocyanin		Phycocyanin				Phycoerythrin I
		*apcC* (L_C_)	*apcE* (L_CM_)	*cpcC* (L_R_)	*cpcD* (L_R_)	*cpcG* (L_RC_)	*cpcL* (L_RM_)	*cpeC* (L_R_)
*Leptolyngbya* JSC-1	T2	1	2	2	1	1	2	1
*Leptothermofonsia* E412	T2	1	2	2	1	1		1
*Ocullathermofonsia* A174	T1	1	2	2	1	3	2	
*Thermoleptolyngbya* A183	T1	1	1	2	1	3	1	
*Thermoleptolyngbya* O-77	T1	1	1	2	1	3	1	
*Trichothermofonsia* B231	T1	1	1	2	1	1		
*Synechococcus* C9	T1	1	1	2	1	2		
*Thermostichus* 60AY4M2	T1	1	2	1	1	1		
*Thermostichus* 63AY4M2	T1	1	1	1	1	1		
*Thermostichus* 65AY6A5	T1	1	2	1	1	1		
*Thermostichus* 65AY6Li	T1	1	2	1	1	1		
*Thermostichus* JA-2-3Ba	T1	1	1	1	1	1		
*Thermostichus* JA-3-3Ab	T1	1	2	1	1	1		
*Thermosynechococcus* PCC 6715	T1	1	1	1	1	3		
*Thermosynechococcus* CL-1	T1	1	1	1	1	3		
*Thermosynechococcus* TA-1	T1	1	1	1	1	3		
*Thermosynechococcus* BP-1	T1	1	1	1	1	3		
*Thermosynechococcus* E542	T1	1	1	1	1	3		
*Thermosynechococcus* NIES-2134	T1	1	1	1	1	3		

Copy numbers of genes are indicated. L_C_, core linker; L_CM_, core–membrane linker; L_R_, rod linker; L_RC_, rod–core linker; and L_RM_, rod–membrane linker.

**Table 4:** Availability of genes encoding putative phycobilin lyases in the genomes of thermophilic cyanobacteria studied.

Species	Pigment Type	Phycocyanin				Phycoerythrin I				
		*cpcE*	*cpcF*	*cpcS*	*cpcT*	*cpeF*	*cpeS*	*cpeT*	*cpeU*	*cpeZ*
*Leptolyngbya* JSC-1	T2	1	1	1	1	1	1	1	1	1
*Leptothermofonsia* E412	T2	1	1	1	1	1	1	1	1	1
*Ocullathermofonsia* A174	T1	1	1	1	1					
*Thermoleptolyngbya* A183	T1	1	1	1	1					
*Thermoleptolyngbya* O-77	T1	1	1	1	1					
*Trichothermofonsia* B231	T1	1	1	1	1					
*Synechococcus* C9	T1	1	1	1	1					
*Thermostichus* 60AY4M2	T1	2	2	1	2					
*Thermostichus* 63AY4M2	T1	2	2	1	2					
*Thermostichus* 65AY6A5	T1	2	2	1	2					
*Thermostichus* 65AY6Li	T1	2	2	1	2					
*Thermostichus* JA-2-3Ba	T1	2	2	1	2					
*Thermostichus* JA-3-3Ab	T1	2	2	1	2					
*Thermosynechococcus* PCC 6715	T1	1	1		1					
*Thermosynechococcus* CL-1	T1	1	1	1	1					
*Thermosynechococcus* TA-1	T1	1	1	1	1					
*Thermosynechococcus* BP-1	T1	1	1	1	1					
*Thermosynechococcus* E542	T1	1	1	1	1					
*Thermosynechococcus* NIES-2134	T1	1	1	1	1					

Copy numbers of genes are indicated.

## Data Availability

The data presented in this study are openly available in the National Center for Biotechnology Information (https://www.ncbi.nlm.nih.gov/genome/, accessed on 1 June 2022).

## Supplementary Materials

The following supporting information can be downloaded at: https://www.mdpi.com/article/10.3390/ijms24065632/s1.
