# Supplementary figures and images for: Characterization of Molecular Diversity and Organization of Phycobilisomes in Thermophilic Cyanobacteria
Source: Int J Mol Sci. 2023 Mar 15;24(6):5632. doi: 10.3390/ijms24065632 (PMC10053587; doi:10.3390/ijms24065632)

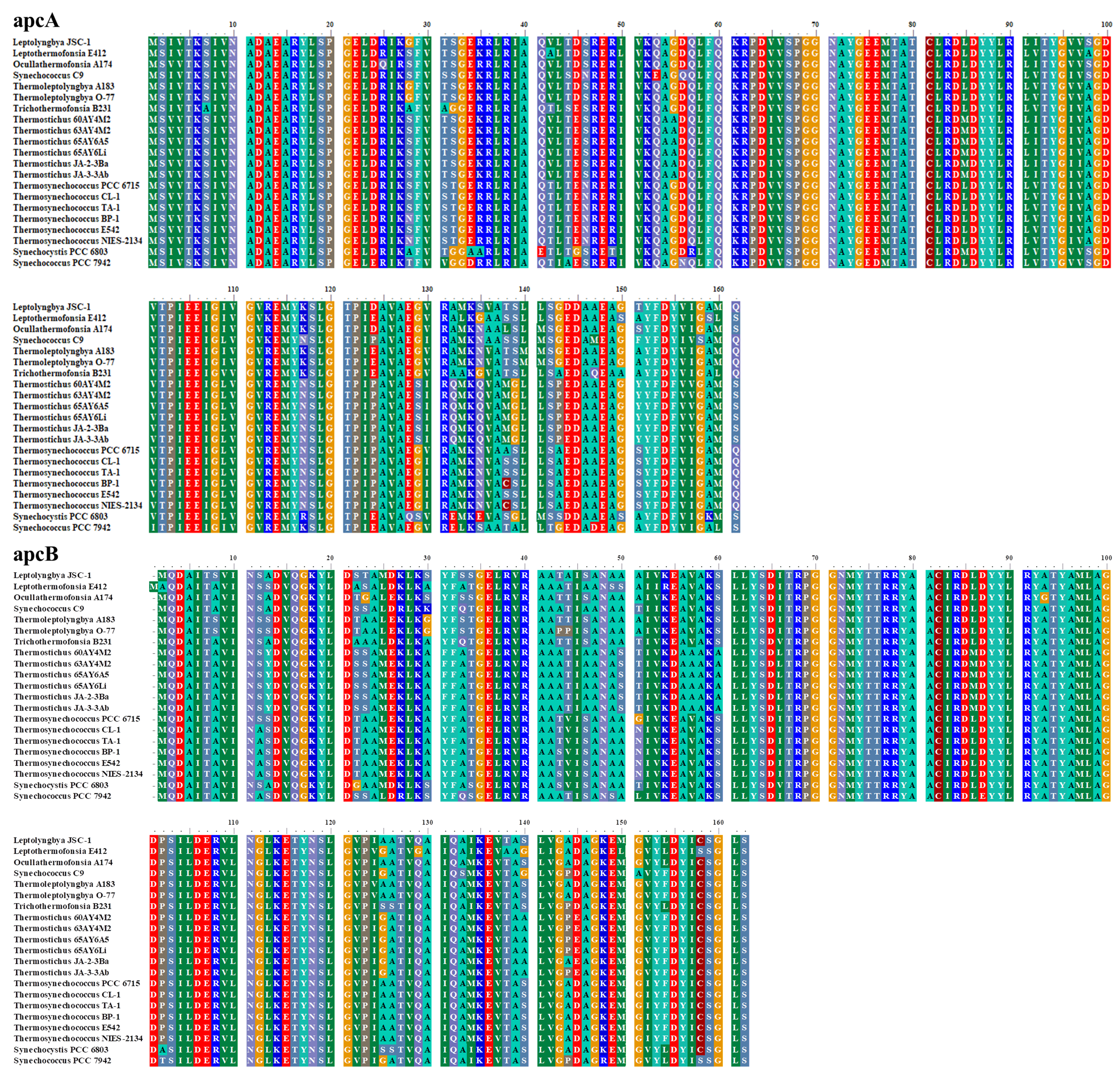

Supplement: Supplementary file 1 [file ijms-24-05632-s001.zip › Figure S1.tif]

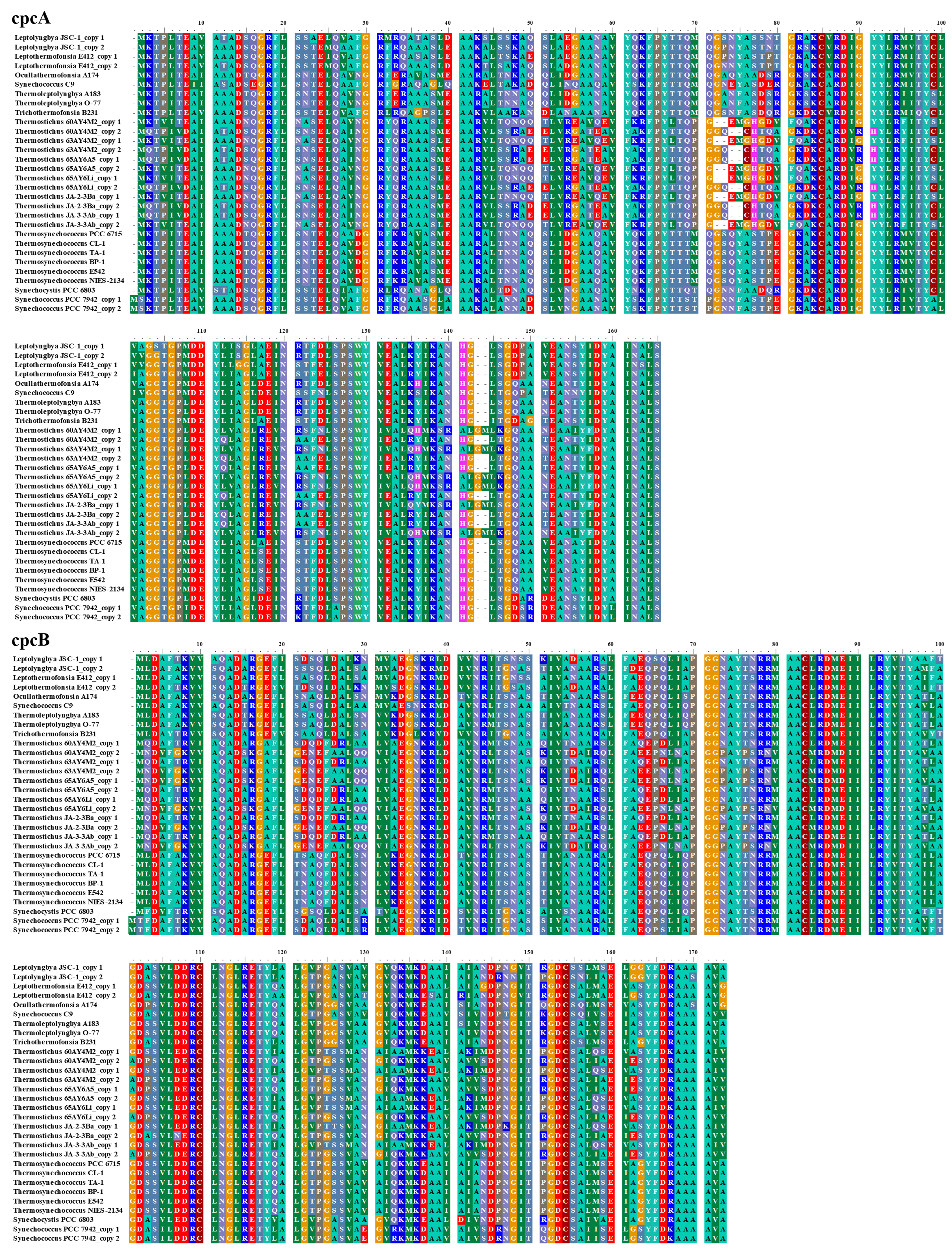

Supplement: Supplementary file 1 [file ijms-24-05632-s001.zip › Figure S2.tif]

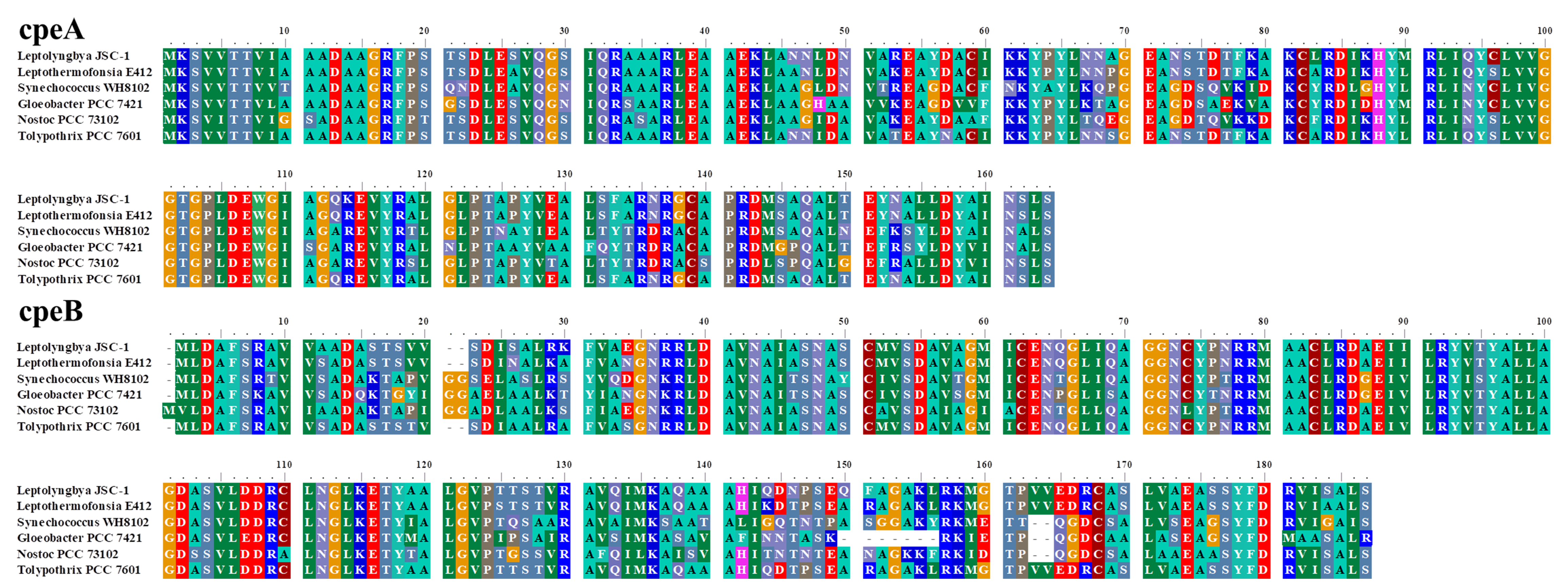

Supplement: Supplementary file 1 [file ijms-24-05632-s001.zip › Figure S3.tif]

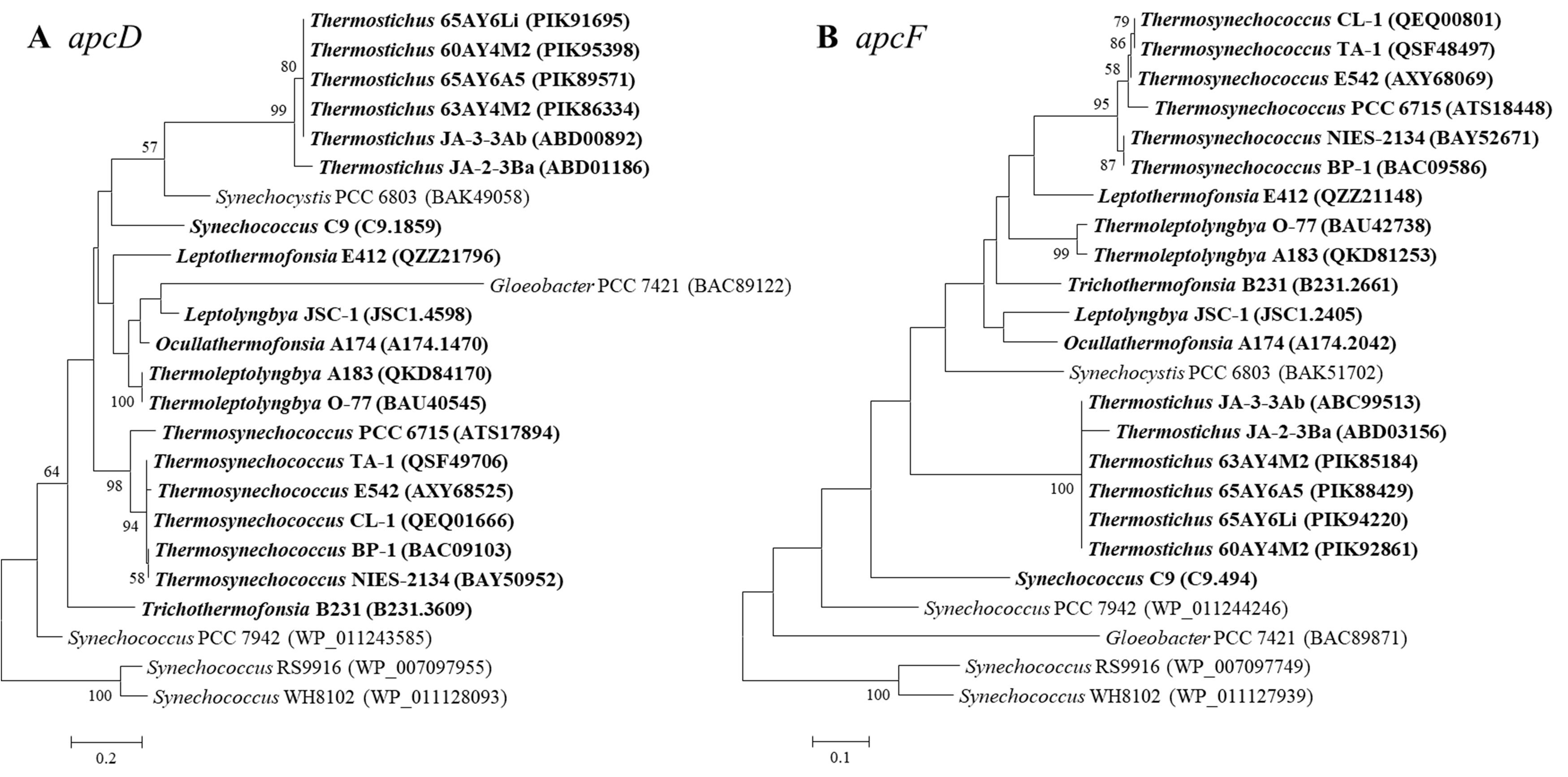

Supplement: Supplementary file 1 [file ijms-24-05632-s001.zip › Figure S4.tif]
